# Sinigrin and Its Therapeutic Benefits

**DOI:** 10.3390/molecules21040416

**Published:** 2016-03-29

**Authors:** Anisha Mazumder, Anupma Dwivedi, Jeanetta du Plessis

**Affiliations:** Centre of Excellence for Pharmaceutical Sciences, North-West University, Private Bag X6001, Potchefstroom 2520, South Africa; anishamajumdar@gmail.com (A.M.); anupma.feb1@gmail.com (A.D.)

**Keywords:** glucosinolates, sinigrin, anticancer, mustard, Brassicaceae family, myrosinase

## Abstract

Sinigrin (allyl-glucosinolate or 2-propenyl-glucosinolate) is a natural aliphatic glucosinolate present in plants of the Brassicaceae family, such as broccoli and brussels sprouts, and the seeds of *Brassica nigra* (mustard seeds) which contain high amounts of sinigrin. Since ancient times, mustard has been used by mankind for its culinary, as well as medicinal, properties. It has been systematically described and evaluated in the classical Ayurvedic texts. Studies conducted on the pharmacological activities of sinigrin have revealed anti-cancer, antibacterial, antifungal, antioxidant, anti-inflammatory, wound healing properties and biofumigation. This current review will bring concise information about the known therapeutic activities of sinigrin. However, the information on known biological activities is very limited and, hence, further studies still need to be conducted and its molecular mechanisms also need to be explored. This review on the therapeutic benefits of sinigrin can summarize current knowledge about this unique phytocompounds.

## 1. Introduction

It is has been well established that natural products are a rich source of compounds for purposes of drug discovery. It is estimated that more than 80% of the world’s population depends on traditional medicine for the treatment of various diseases [1]. During the past decade, it was discovered that many classes of secondary metabolites, such as glucosinolates and their hydrolysis products, have crucial bioactive(s), can be utilized as nutraceuticals, and also have therapeutic benefits [2,3]. The biological actions are dependent on the levels and classes of glucosinolates present [4]. Glucosinolates are a class of abundant secondary metabolites characteristic of the plants of the mustard family (Brassicaceae). Glucosinolates are broken down enzymatically by myrosinase, mainly into isothiocyanates, cyanides and thiocyanates which are the main bioactives, responsible for pharmacological effects [5]. Numerous members of the Brassicaceae family have been commercialized globally, for animal and human consumption, as a rich source of nutrients and healthy products [6]. Mustard, classified to the Brassicaceae family, is extensively consumed by humans all over.

**Figure 1:**
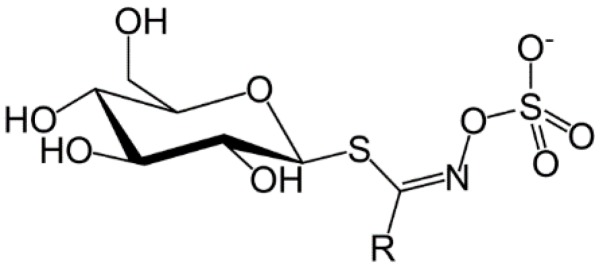
Structure of glucosinolates, where R is the variable side chain from amino acids [9].

The first observations on the unique properties of glucosinolates and isothiocyanates (mustard oils) were indicated in the early 17th century and attempts were made to understand the chemical origin of the strong taste of mustard seeds. In 1959, Challenger reviewed the discovery and early history of glucosinolates as well as the involvement of the enzyme myrosinase (β-thioglucosidase) in their conversion to isothiocyanates [7]. Glucosinolates contains a β-d-thioglucose group linked to a sulfonated aldoxime moiety and a variable side chain derived from amino acids (Figure 1). Glucosinolates are a class of water soluble compounds as a result of their ionized sulfate and hydrophilic thioglucose moieties and owing to their physiological properties they are not easily separated and purified [8].

Glucosinolates have been reported to exhibit different pharmacological properties, such as antifungal, antibacterial, bioherbicidal, antioxidant, antimutagenic, anticancer, and anti-inflammatory, *etc.* Sinigrin is one of the glucosinolates of which the bioactivity should be explored and its known activity enhanced through optimal delivery to the human body. A number of studies have been performed on the therapeutic activities of sinigrin and revealed its anticancer, anti-inflammatory, antibacterial, antifungal, antioxidant, and wound healing effects. It is believed that the metabolic activation of sinigrin leads to the formation of isothiocyanates, which are responsible to contribute to the anti-tumor effects and other biological actions.

**Figure 2:**
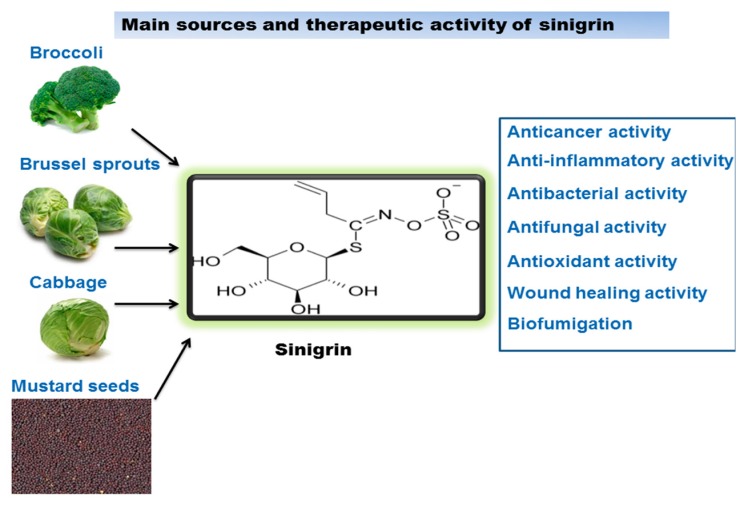
Sinigrin sources and its therapeutic activity.

Sinigrin is a major glucosinolate, associated with the family of glucosides present in the Brassicaceae family, such as the seeds of black mustard (*Brassica nigra*), brussels sprouts, and broccoli. It has been reported that *Brassicaceae juncea* (Indian mustard) contains significant amounts of sinigrin. Since ancient times mustard has been used as a food and has illustrated medicinal benefits in Ayurveda. The Ayurvedic tradition established mustard as a valuable herb which has therapeutic effects. For thousands of years Indian mustard seeds and its oil have been aptly used to relieve joint pain, fever, alleviate cough and colds, lessened swelling, and in cleaning the cranial cavity. Mustard oil has also been used for the treatment of various skin diseases and wounds [10]. Scientific investigations have encouraged us to exploit this potential in a very effective manner. Sinigrin is known as the precursor of the myrosinase–mediated breakdown product allyl isothiocyanate, which exerts various biological effects and also has a vital role in the prevention of cancer and DNA damage caused by carcinogens [11]. They have also been exploited as nutritional supplements for their preventive and medicinal benefits on various different diseases. In a study, sinigrin showed to reduce the level of plasma triglyceride, hence suggesting that alkenyl glucosinolates could be an encouraging agent to prevent postprandial hypertriglyceridemia [12]. Figure 2 illustrates the main sources of sinigrin and gives a summary of its biological activity. In this review the therapeutic activities, such as anticancer, anti-inflammatory, antibacterial, antifungal, antioxidant, wound healing effects, and biofumigation of sinigrin have been discussed.

## 2. Extraction of Sinigrin

The isolation and separation of glucosinolates is an extremely arduous task, due to their physicochemical properties. The existence of the sulfate group and of the thioglucose moiety results in a very low octanol–water partition coefficient (log Po/w) to fall in the low value domain, hence proposing that these classes of compounds are very hydrophilic and mostly water-soluble [13]. Indian mustard seeds contain high amounts of sinigrin compared to the other plants of the Brassicaceae family, thus making it one of the most suitable raw materials for large scale extraction [14]. Various extraction methods of glucosinolates have been demonstrated, including boiling water extraction [15,16] and aqueous organic solvent extraction. Different parameters, such as solvent composition, particle size, temperature, and the number of required extraction steps, were optimized utilizing pressurized liquid extraction and analysis by electrospray ionization mass spectrometry in the negative ion mode [17].

To enhance the yield of extraction of sinigrin, it is important to optimize the extraction process. Several methods of extraction of sinigrin have been endeavored. The ultrasonic–stimulated solvent extraction method was noted to be promising in improving the productivity of sinigrin [18]. Four extraction techniques for sinigrin from Centennial (*Brassica juncea* L.) seeds, were compared; namely boiling water, boiling 50% (*v*/*v*) water/acetonitrile, and 100% methanol, 70% (*v*/*v*) aqueous methanol at 70 °C. It was found that 50% (*v*/*v*) water/acetonitrile was the most efficient extraction solvent [19]. A cold water extraction method was developed for extraction of sinigrin from *Brassica juncea*. Sinigrin has been positively identified by using (1) ^1^H-NMR spectroscopy [20]. A hollow fiber microdialysis sampling, coupled to ion pair liquid chromatography, was developed for the direct determination of sinigrin without desulfation [21].

To identify sinigrin in traditional Chinese medicine, and also determine the quality thereof, a near-infrared diffuse reflectance spectroscopy method was employed [22]. Parent ion mapping analytical mass spectrometry was utilized to detect glucosinolate sinigrin [23]. A reverse-phase HPLC method was developed for the determination of sinigrin and other various glucosinolates in traditional Chinese plants [24] and their detection was performed by a quadrupole time-of-flight tandem mass spectrometer.

## 3. Therapeutic Benefits of Sinigrin 

### 3.1. Anticancer Activity 

The potential of sinigrin to prevent the growth of cancer cells have been well established. Allyl isothiocyanate-rich mustard seed powder (MSP-1), was stably stored as its glucosinolate precursor (sinigrin) in MSP-1. On addition of water, sinigrin was readily hydrolyzed by endogenous myrosinase. Sinigrin, itself, was not bioactive, but hydrated MSP-1 caused apoptosis and G_2_/M phase arrest in bladder cancer cell lines *in vitro*. In an orthotopic rat bladder cancer model, it inhibited bladder cancer growth and blocked muscle invasion [25]. The Jie research group studied the anti-proliferative activities of sinigrin in a model of carcinogen-induced hepatotoxicity in rats. It was found that sinigrin significantly inhibited the proliferation of liver tumor cells and the number of surface tumors in the rat liver was lessened. Sinigrin also induced apoptosis of liver cancer cells through up-regulation of the p53 and down-regulation of the Bcl-2 family members and caspases. Their findings indicated that the liver functions were gradually restored after treatment with sinigrin and it did not cause any liver toxicity. Cell cycle analysis showed that sinigrin caused cell cycle arrest in the G0/G1 phase. The results illustrated that sinigrin displayed anti-proliferative activity in carcinogen-induced hepatocarcinogenesis in rats and indicated the potential of sinigrin as an anti-cancer agent against liver cancer [26].

In another study, Ethiopian mustard (*Brassica carinata* A. Braun) and its glucosinolate sinigrin were tested in the *in vitro* HL60 (human promyelocytic leukaemia cell line) and *in vivo Drosophila melanogaster* systems to determine the anti-mutagenic and anti-proliferative properties. The antitumor activity of the *B. carinata* and, its major glucosinolates, sinigrin was determined by measuring the relative inhibitory capacity of tumors growing in HL60 cells. *B. carinata* showed a dose-response curve with a high tumoricide activity in HL60 cells (IC_50_ value of 0.28 mg·mL^−1^). Single sinigrin to the cell medium did not produce cytotoxic effects. When sinigrin was hydrolyzed by the enzyme myrosinase by addition to it, exhibited anti-proliferative activity (hydrolyzed sinigrin IC_50_ = 2.71 μM) [27]. They also studied the anti-genotoxicity and the results obtained contributed to the health properties of *B. carinata* and sinigrin in DNA protection. The percentage of inhibition of *B. carinata* and sinigrin when are assayed against H_2_O_2_. The addition of plant samples to the fly food produced anti-mutagenic effects. Both of them showed a high desmutagenic and recombinogenic potency. The lowest concentrations assayed for *B. carinata* plant samples showed more anti-genotoxic effects than those of higher (78.46% clone inhibition). For sinigrin, the highest anti-genotoxic effect displayed percentage of inhibition of clone formation of 84.61%.

Investigations were conducted on the inhibition of dimethylhydrazine-induced aberrant crypt foci and induction of apoptosis in the rat colon, following oral administration of the glucosinolate sinigrin [28] to rats for three months. An increase in apoptosis in colonic crypts was displayed, when exposed to the carcinogen. There was no significant induction of apoptosis in rats when sinigrin, alone, was fed; however, sinigrin administered after dimethylhydrazine suppressed the induction of aberrant crypt foci. This may be due to increased apoptotic deletion of damaged stem cells in the crypts of rats with treated sinigrin.

The activity of sinigrin indole-3-carbinol (I3C) on DNA methylation in target tissues of tobacco-specific nitrosamine 4-(methylnitrosamino)-1-(3-pyridyl)-1-butanone (NNK) tumorigenesis, and also the effect of dietary sinigrin on NNK tumorigenicity were assessed in a two-year bioassay in F344 rats [29]. The study reported that sinigrin decreased 7-methylguanine formation in hepatic DNA, but had no effect on 7-methylguanine levels of lung or nasal mucosa DNA. I3C increased 7-methylguanine levels in hepatic DNA, but decreased DNA methylation in lung and nasal mucosa. The bioassay, suggested that sinigrin had no effects on NNK tumorigenesis in the target tissues, but sinigrin plus NNK displayed significant incidence of pancreatic tumors than in the NNK treated alone. This study concluded that, absence of any inhibitory effect of sinigrin on NNK hepatic cells tumorigenesis, could be due factors other than DNA methylation and O6-methylguanine repair which can be considered in evaluating the effects of dietary compounds on NNK hepatic tumorigenesis, and also stated that the contrary effects on NNK-induced hepatic DNA-methylation by sinigrin and I3C shows the complexities of dietary modulation of carcinogenesis.

In another study, the effects of sinigrin and indole-3-carbinol (I3C) on the hepatocarcinogenesis induced by diethyl-nitrosamine (DEN) were studied in male ACI/N rats. When rats where treated with diethyl-nitrosamine and diet containing 1200 ppm. Sinigrin and rats fed with rats where treated with diethyl-nitrosamine and diet containing 1000 ppm. (I3C), the incidences of iron-excluding altered foci, liver cell tumors, and the tumor multiplicity were significantly smaller than when rats when treated only with diethyl-nitrosamine. This study suggested that sinigrin and indole-3-carbinol inhibited the hepatocarcinogenesis induced by diethyl-nitrosamine when delivered concurrently with the carcinogen [30].

The inhibitory effects of indole-3-carbinol (I3C) and sinigrin during initiation and promotion phases of 4-Nitroquinoline 1-oxide-induced rat tongue carcinogenesis were demonstrated in male ACI/N rats. Both I3C and sinigrin treated during initiation and post initiation phase suppressed preneoplastic and neoplastic lesions of the tongue epithelium induced by 4-nitroquinoline-1-oxide. It also caused significant decreased in the number and area of silver-stained nucleolar organizer regions protein, they are known as indices of cell proliferation effects. The results indicated that I3C and sinigrin inhibited rat tongue carcinogenesis in both the initiation and post initiation phases, followed by treatment with 4-nitroquinoline-1-oxide [31]. They suggested that the mechanism by which by which I3C and sinigrin displayed their inhibitory actions on tongue carcinoma was not clear but it could be related to the actions these compounds on the metabolic activation, formation of DNA adduct, detoxification of 4-nitroquinoline-1-oxide or formation of radicals.

Few speculations for the mechanisms of the anti-carcinogenesis activity of glucosinolates have been recognized. The mechanism of the anti-carcinogenesis activity of glucosinolates is unknown [32]. Blocking effects are believed to involve modulation of enzymes, which can reduce exposure of target tissues to DNA damage. Isothiocyanates have shown to induce the activity of phase II enzymes, including glutathione *S*-transferase and quinone reductase, in the small intestinal mucosa and liver, and also to block chemical carcinogenesis. Increased consumption of brassica vegetables induces glutathione *S*-transferase in humans and increased protection against cancer [33]. Another speculated mechanism of anti-carcinogenesis is suppression of tumor development following the initiation of pre-cancerous cells. Other mechanisms of suppression are the deletion of initiated cells from genetically-damaged tissue by apoptosis [34].

Recently, our research group tested the effect of sinigrin on melanoma cells (A-375) and in normal human keratinocytes (HaCaT) [35]. We also investigated the use of a vesicular carrier system called phytosomes, to encapsulate sinigrin and determine whether the phytosome complex could enhance the effects of sinigrin on melanoma cells. Our results indicated that sinigrin alone, at higher concentration, displayed about 46% toxicity, whilst the sinigrin-phytosome complex inhibited cytotoxicity by 74%. These findings suggested that the sinigrin-phytosome complex did enhance the cytotoxic effects of sinigrin. It was noteworthy that sinigrin and its phytosome complex displayed minimal toxicity towards HaCaT cells. When the anticancer effects of sinigrin were studied by other researchers, it was noticed they exhibited strong anticancer activity. Although the phytosome formulation increased its activity at higher concentration, sinigrin did not exhibit much cytotoxic effect towards A-375 melanoma cells. 

### 3.2. Anti-Inflammatory Activity

Sinigrin’s effect on the production of inflammatory mediators in lipopolysaccharide (LPS)-activated RAW 264.7 macrophages, have been examined by Lee [36]. They investigated the anti-inflammatory effects of sinigrin on nitrite oxide (NO) and pro-inflammatory cytokine production by utilizing colorimetric and ELISA assay. By using Western blot assays the researchers also examined the expression of MAPK, NLRP-3, and p65. The results indicated that sinigrin did not reduced the NO production, but sinigrin inhibited the levels of tumor necrosis factor-α (TNF-α) and interleukin-6 (IL-6). Sinigrin blocked phosphorylation of JNK and p38, but not ERK. Sinigrin treatment significantly suppressed the expression of p65 and NLRP-3. These results revealed sinigrin has potential anti-inflammatory activity, which may result from the inhibition of MAPK phosphorylation, expression of NLRP-3 and p65, and also lowers the production of pro-inflammatory mediators. 

The effectiveness of sinigrin against atherosclerosis (chronic inflammatory disease) in ApoE-deficient mice was studied. Sinigrin exhibited significant repressive effects on the expression of VCAM-1 and ICAM-1 in ApoE mice. It also mitigated the level of oxLDL, HDL, LDH, triglyceride, and cholesterol in serum. The serum levels of sterol-regulatory element binding protein-2 (SREBP-2), oxidized low-density lipoprotein receptor-1 (LOX-1), and liver X receptors (LXRs) were reduced by sinigrin; it also decreased the serum levels of IL-6 and TNF-α. It can, therefore, be concluded that sinigrin has anti-atherosclerotic activity [37]. 

Lee and Lee studied the effect of sinigrin on the expression of the vascular cell adhesion molecule-1 (VCAM-1) in TNF-α-induced vascular smooth muscle cells (VSMCs). The data suggested that sinigrin suppressed the nuclear translocation of NF-κB induced by TNF-α. Through the suppression of NF-κB signaling pathways, sinigrin inhibited the TNF-α-stimulated VCAM-1 expression [38]. The result from the two above studies, it can be concluded that sinigrin acts as an anti-atherosclerosis therapeutic agent.

### 3.3. Antibacterial Activity

Glucosinolate hydrolysis products are potent inhibitors of bacterial activity. Sinigrin is not usually antimicrobial; when it is enzymatically hydrolyzed to form allyl isothiocyanate it exhibited potent antimicrobial activity against food spoilage and pathogenic organisms [39,40]. Allyl isothiocyanate showed minimum inhibitory concentrations as 25 µL/L at pH 4.5 with greater antimicrobial activity at low pH value than at high pH 8.5 against *Escherichia coli* O157:H7. This indicated a gradual reduction of the antimicrobial activity when the pH was raised. Hence, it was suggested that allyl isothiocyanate could work better in more acid foods. The speculated mechanism of the antimicrobial activity of isothiocyanates could be related to intracellular inactivation of sulphydryl-enzymes; this was concluded from the observations, where proteins and sulphydryl compounds were able to suppress the antimicrobial effects of diverse isothiocyanates [41]. The thioredoxin system is known for its essential role in DNA synthesis. It has shown the capability of allyl isothiocyanate in crossing the plasma membrane and reaching the cytoplasm of prokaryotic and eukaryotic cells [42]. Hence, suggesting that the antibacterial activity of allyl isothiocyanate could be related to the inhibition of DNA synthesis. Allyl isothiocyanate is known to inhibit the catalysis of thioredoxin reductase and acetate kinase, which are responsible for important metabolic reactions in bacteria. Thus, it can be proposed that allyl isothiocyanate have many targeted antimicrobial activity, as they can cause enzymatic inhibition and membrane damage [43].

The antimicrobial activity of residual endogenous plant myrosinase in Oriental and yellow mustard powders and a deoiled meal (which contained more glucosinolate than unextracted mustard powder of each type of mustard), against *E. coli* O15:H7 during dry-fermented sausage ripening was investigated. It was noticed that when 4% (*w*/*w*) deodorized yellow mustard powder containing myrosinase from hot mustard was added to the sausages, ripening required between 18 and 24 d to reduce *E. coli* O157:H7 numbers. The 2% (*w*/*w*) deoiled yellow mustard meal treatment containing myrosinase activity was as potently antimicrobial as 4% yellow mustard powder and took 21 d to obtain the reduction. A significant difference in bactericidal activity was noticed between yellow and Oriental mustard treatments, where yellow mustard was more antimicrobial. This may be due to the yellow mustard contained higher glucosinolate levels than Oriental mustard. It was believed that myrosinase activity contributed to the high antimicrobial actions of mustard when used in sausage against *E. coli* O157:H7 through its hydrolysis of glucosinolates [44]. 

In a study by Herzallah and Holley, evaluated the use of carboxymethyl cellulose (CMC) nanoparticulate on the antimicrobial activity of CMC films containing sinigrin against *E. coli* O157:H7 on fresh beef. The study indicated that the films with nanoparticulation that contain sinigrin in oriental mustard significantly exhibited more antimicrobial activity than films without nanoparticulation. Hence it was concluded that the nanopartiulation of CMC significantly enhanced the antimicrobial effects of the films having sinigrin [45].

Sinigrin and its degradation products, such as allyl isothiocyanate, allyl cyanide (AC), 1-cyano-2,3-epithiopropane (CETP), and allyl thiocyanate (ATC), were tested for antibacterial activity on nine species of bacteria and eight species of yeasts. The results gained from this study indicated that sinigrin, AC, and CETP at 1000 ppm were not inhibitory to any bacteria or yeast growth and allyl isothiocyanate was the most inhibitory of the sinigrin hydrolysis products [46]. 

Brabban and Edwards reported that sinigrin was found to be innocuous to all the organisms being tested but its hydrolysis products exhibited inhibitory effects of growth [47]. In this investigation rapemeal, containing potentially toxic compounds; glucosinolates, was examined as a substrate for the growth of micro-organisms. Before its hydrolysis the initial inhibitory sinigrin concentration was found to be species-dependent with *Bacillus subtilis* being the most resistant (80 μg·mL^−1^) and *Saccharomyces cerevisiae* (40 μg·mL^−1^) the most sensitive one. Three Gram-positive organisms tested were found to be more resistant to hydrolysis products than other micro-organisms. It was observed in rapemeal media growth inhibition was dependent on the glucosinolate content of the rapemeal.

In an investigation by Lara-Lledo and their group, the ability of *Listeria* monocytogenes to convert glucosinolates present in deodorized oriental and yellow mustard, as well as pure sinigrin, into their respective isothiocyanates during *in vitro* study and on sliced bologna vaccum-packed with polyvinyl polyethylene glycol graft copolymer packaging films. During broth tests with deodorized (myrosinase-inactivated) mustard extracts or with purified sinigrin inhibition was only displayed when exogenous myrosinase was added. It was noticed that when pure sinigrin, oriental or yellow mustard extracts were incorporated in films containing 3%, 5%, and 6% (*w*/*w*) of the corresponding glucosinolate and used to package bologna inoculated with L monocytogenes., the pathogen was found in bologna packed with the oriental mustard extract. The yellow mustard extract exerted less inhibition and the pure sinigrin did not show antimicrobial activity [48].

### 3.4. Antifungal Activity

The potential of members of the Brassicaceae family have been shown to produce significant quantities of antifungal compounds in roots [49]. The results indicated the glucosinolate-mediated resistance to fungi in the roots of *Brassica* species. Investigations by Ocampo, have indicated that extracts from the roots of various *Brassica* species and reactions of the glucosinolate sinigrin with myrosinase inhibited the germination of *Glomus mosseae* spores [50]. 

The fungitoxicity of allyl-isothiocyanate vapour against *Penicillium expansum,* the agent of blue mould on pears, was studied and it was observed that the use of allyl-isothiocyanate produced from pure sinigrin (*Brassica juncea*) was effective and an alternative to synthetic fungicides against *P. expansum* [51].

### 3.5. Antioxidant Activity

The Ippoushi research group have demonstrated the sinigrin antioxidant activity [52]. It is known that allyl isothiocyanate is produced from sinigrin and this suppresses nitric oxide production and the induction of inducible nitric oxide synthase in lipopolysaccharide-activated J774.1 macrophages. Myrosinase, in cruciferous vegetables, is not activated at the time of processes of cooking and, therefore, cannot produce allyl isothiocyanate from sinigrin, thus sinigrin is mainly taken by human beings and not allyl isothiocyanate. 

They tried to demonstrate the *in vivo* suppressive effect of sinigrin administration on nitric oxide formation induced lipopolysaccharide administration. Their study was performed to assay the levels of urinary nitrate + nitrite and allyl isothiocyanate in rats. The results indicated that the intake of sinigrin significantly reduced urinary levels of nitrate + nitrite, an index of nitric oxide production in lipopolysaccharide treated rats. It also revealed that sinigrin has antioxidative properties and lowers the level of reactive nitrogen species. Generally reactive oxygen species and reactive nitrogen species are known to be involved in the multistage carcinogenesis process [53].

### 3.6. Wound Healing Activity

The wound healing properties of sinigrin were not previously studied. Our research group recently, for the first time, revealed that sinigrin has the potential to also cure wounds [35]. The *in vitro* wound healing activity was tested on normal human keratinocytes (HaCaT). Sinigrin was also formulated using a vesicular system called phytosome. The findings suggested that the sinigrin-phytosome complex enhanced the wound healing actions of sinigrin. The effects of sinigrin and its phytosome formulations were studied at two different concentrations. It was observed that, at lower concentration of 0.07 mg/mL, the sinigrin–phytosome complex displayed 79%, whilst sinigrin showed only 50% of wound closure. At the higher concentration 0.14 mg/mL the sinigrin–phytosome complex completely cured the wound (100%), whereas the sinigrin alone displayed only 71% wound healing. These results confirmed the wound healing activity of sinigrin, which was also augmented by encapsulation in the phytosome delivery system. 

### 3.7. Biofumigation

Glucosinolates and their breakdown products, like isothiocyanates, have attained attraction as possibility of using them as natural “pesticides” in a process called biofumigation, that helps to reduce soil-borne pests and pathogens by placing plants of the Brassicales order into the soil [54]. The reactive isothiocyanates are formed enzymatically from glucosinolates and after tissue disruption glucosinolates are hydrolyzed by thioglucosidase (myrosinase), a β-d-thioglucosidase that cleaves “β-d-glucose. The yielded aglycone degrades to form isothiocyanates, thiocyanates, nitriles, epithionitriles, and oxazolidine 2-thione [55,56]. However, the non-enzymatic thermal degradation of glucosinolates could produce chemical species identical to products of enzymatic hydrolysis [57]. The breakdown products are relatively small molecules making many of them volatiles and have been shown to behave as attractants for insects for seeking food or egg-laying sites rather than acting as a direct insecticide. Enzymatic decomposition of allyl glucosinolate (sinigrin) breaks down in soils to allyl isothiocyanate and allyl cyanide, allyl nitrile and these breakdown products and glucosinolate-containing plant tissues have been utilized in controlling soil-borne plant pests [58].

Lethality test using *Caenorhabditis elegans* was used to assess toxicity of glucosinolates and their enzymatic breakdown products. It was observed that in the absence of the enzyme thioglucosidase (myrosinase) sinigrin was found to be nontoxic at all concentrations, but addition of thioglucosidase increased toxicity by two orders of magnitude [59], thus suggesting it can be used as an effective nematicide.

In a study by Pratt research group, aphids were supplied as a food source to two species of polyphagous ladybird, *Adalia bipunctata* and *Coccinella septempunctata*. When *Brassica nigra* diets containing 0.2% sinigrin, was fed to First instar *A. bipunctata*, it was unable to survive, but when fed aphids reared on diets having 0% sinigrin, it was observed that the survival rates were higher. First instar *Coccinella septempunctata* survived when fed with aphids reared on *Brassica nigra* having up to 1% sinigrin. It was noteworthy that the presence of sinigrin in the aphid diet decreased the growth of larvae and increased the time period needed for larvae to reach second instar for this species of ladybird. These results indicated that the presence of sinigrin in the diet of *Brassica nigra* makes this aphid not suitable as a food source for *Adalia bipunctata* [60].

The uptake of a glucosinolate (sinigrin) was investigated when aphids fed on plants. In nymphs of the wingless aphid morph, glucosinolate levels continued to increase throughout the development to the adult stage, but the quantity in nymphs of the winged form peaked before eclosion and then declined. Winged aphids excreted significantly higher amounts of glucosinolate in the honeydew in comparison to wingless aphids. The study suggested that the higher level of sinigrin in wingless aphids had a highly negative impact on survival of a ladybird predator. Larvae of *Adalia bipunctata* were not able to survive when a 1% sinigrin diet was fed to adult wingless aphids. It survived successfully when fed aphids from a glucosinolate-free diet [61].

## 4. Conclusions

In summary, not much information has been published that acknowledges the therapeutic potential of sinigrin. In this review, we have shed some light on the importance of this unique compound with its numerous known biological activities. We have discussed sinigrin’s anti-cancer, anti-inflammatory, antibacterial, antifungal, antioxidant, and wound healing effects, and biofumigation. However, the number of studies which proves these biological activities is less in number and more studies are required to confirm and further investigate these activities. 

More studies are needed to further explore the still-unknown activities, and also the mechanism of action, of *sinigrin* by which it wields its therapeutic effects. Furthermore, promising investigations in the area of drug carrier systems can be a useful approach to enhance the therapeutic activities of sinigrin.
